# Mulberry Twig Alkaloids Improved the Progression of Metabolic-Associated Fatty Liver Disease in High-Fat Diet-Induced Obese Mice by Regulating the PGC1α/PPARα and KEAP1/NRF2 Pathways

**DOI:** 10.3390/ph17101287

**Published:** 2024-09-27

**Authors:** Mengqing Zhang, Chengcheng Guo, Zonglin Li, Xiaoling Cai, Xin Wen, Fang Lv, Chu Lin, Linong Ji

**Affiliations:** Department of Endocrinology and Metabolism, Peking University People’s Hospital, Beijing 100044, China

**Keywords:** MAFLD, mulberry twig alkaloids, PGC1α, NRF2, lipid metabolism, anti-oxidative stress

## Abstract

**Background/Objectives**: Metabolic-associated fatty liver disease (MAFLD) is one of the most common liver disorders associated with obesity and metabolic syndrome, and poses a significant global health burden with limited effective treatments. The aim of this study was to assess the protective effects of mulberry twig alkaloids (SZ-A) on MAFLD and to further investigate the underlying mechanisms including the specific targets or pathways. **Methods**: Diet-induced obesity (DIO) and normal mouse models were established by feeding C57Bl/6J mice with a high-fat diet (HFD) or common diet for 12 weeks. SZ-A, dapagliflozin, and placebo were administered to corresponding mouse groups for 8 weeks. Data of fasting blood glucose, glucose tolerance, insulin tolerance, and the body weight of mice were collected at the baseline and termination of the experiment. Serum liver enzymes and lipids were measured by ELISA. Western blotting, qPCR, and pathological section staining were implemented to evaluate the degrees of liver steatosis, fibrosis, and oxidative stress in mice. **Results**: In DIO mouse models, high-dose SZ-A (800 mg/kg/d) treatment significantly inhibited HFD-induced weight gain, improved insulin tolerance, and reduced serum alanine aminotransferase, total cholesterol, and triglyceride levels compared with placebo. In DIO mice, SZ-A could alleviate the pathological changes of hepatic steatosis and fibrosis compared with placebo. Lipid catabolism and antioxidant stress-related proteins were significantly increased in the livers of the high-dose SZ-A group (*p* < 0.05). Inhibition of PGC1α could inhibit the function of SZ-A to enhance lipid metabolism in hepatocytes. PGC1α might interact with NRF2 to exert MAFLD-remedying effects. **Conclusions**: By regulating the expression of PGC1α and its interacting KEAP1/NRF2 pathway in mouse liver cells, SZ-A played important roles in regulating lipid metabolism, inhibiting oxidative stress, and postponing liver fibrosis in mice with MAFLD.

## 1. Introduction

Metabolic-associated fatty liver disease (MAFLD) originates from metabolic stress-related liver injury, which is strongly associated with insulin resistance and relevant genetic susceptibility [1,2]. MAFLD, as a disease with similar prevalence to obesity, could potentially progress to nonalcoholic steatohepatitis, liver fibrosis, and eventually cirrhosis and hepatocellular carcinoma (HCC) [3,4]. In the last decade, a growing body of studies has demonstrated that the disability-adjusted life years associated with MAFLD increased greatly from countries with low to moderate sociodemographic indices [5]. The prevalence of MAFLD has also risen sharply in China in recent years, in which it affects over 30% of the total population and has exceeded twice the prevalence in Western countries [5]. The surge in the incidence of MAFLD poses a huge disease burden worldwide, and more effective ways to treat MAFLD still await further refinement.

The primary pathogenesis of MAFLD was considered to be lipid deposition and oxidative stress in the liver, where hyperglycemia would aggravate this process [6,7,8]. When the lipid content surpasses the normal regulatory load of the body due to chronic over-intake, the balance between liver lipid production and catabolism is disturbed, and free fatty acids (FFAs) would then accumulate in the liver. These deposited lipids serve as substrates to produce toxic lipid metabolites that induce endoplasmic reticulum (ER) stress, mitochondrial dysfunction, oxidative stress, cellular damage, and cytokine release, which can result in liver injuries and therefore instigate MAFLD [9].

Mulberry twig alkaloids (known as Sangzhi-alkaloid in Chinese, hereafter referred to as SZ-A) is extracted from Mulberry Ramulus. It has demonstrated effectiveness in managing type 2 diabetes (T2D) in China [10,11]. The main components are 1-deoxynojirycin (1-DNJ), fagopycin (FA), and 1,4 dideoxy-1,4-iminod-arabinitol (DAB) [12]. SZ-A has a highly selective and precise inhibitory effect on intestinal glycosidases, and in vitro experiments have confirmed that its inhibitory effect on disaccharidase activity is stronger than or equivalent to acarbose [12]. In addition to the regulatory effects on glucose metabolism, SZ-A could also reduce the inflammatory response to a certain extent by blocking the activation of the p38 mitogen-activated protein kinase (p38 MAPK), extracellular signal-regulated kinase (ERK), and stress-activated protein kinase (c-Jun N-terminal kinase, JNK) signaling pathways [13]. Regarding lipid metabolism, previous studies have demonstrated that SZ-A could lower serum lipids through multiple mechanisms including reducing body weight, improving overall energy metabolism, and enhancing lipid consumption in hepatocytes [10]. Since the progression of MAFLD is closely associated with glucolipid metabolism disorder (mostly dyslipidemia) and inflammation [6,7,8], SZ-A may exert potential therapeutic effects for MAFLD. In previous research, it was observed that SZ-A ameliorated the progression of MAFLD in mice with high-fat diet (HFD) [10]. However, the specific regulatory mechanisms by which SZ-A relieves MAFLD have not been fully revealed. Whether SZ-A introduces liver benefits through interacting with other potential targets in multiple tissues and organs throughout the body still needs exploration.

Therefore, the aim of this study was to evaluate the therapeutic effects of SZ-A on MAFLD and to investigate its mechanism of action in depth, providing further reference for the treatment of MAFLD in the future.

## 2. Results

### 2.1. High-Dose SZ-A Decreased Body Weight and Improved Insulin Tolerance in Mice Fed with HFD

**Figure 1:**
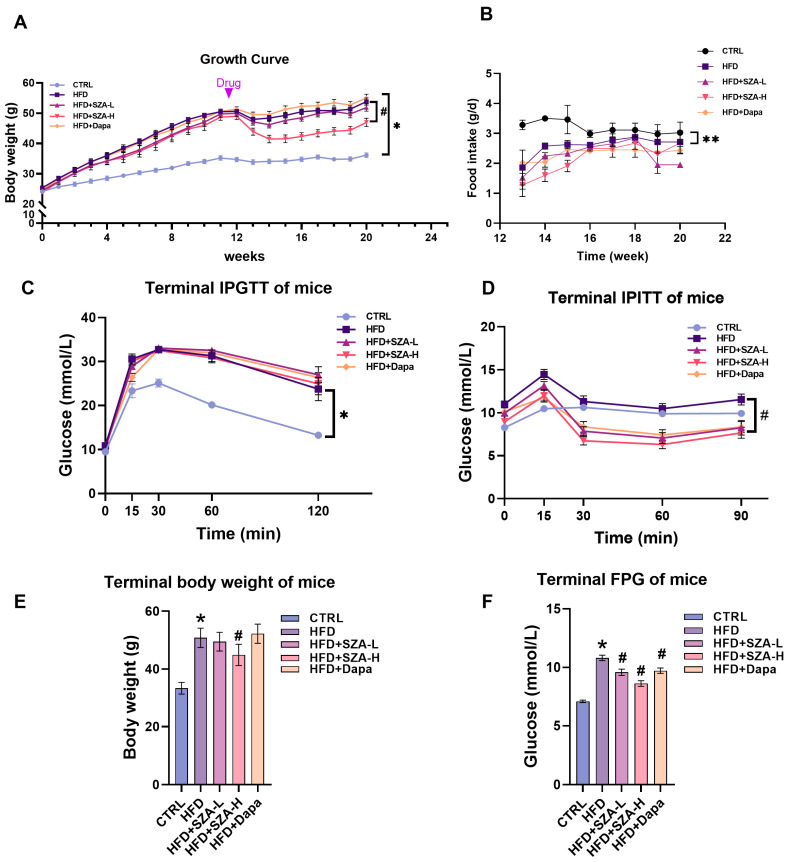
The figure presents a comprehensive analysis of the impact of Sangzhi alkaloids (SZA) and dapagliflozin on mice subjected to a high-fat diet (HFD). (**A**) illustrates the growth curve, detailing the average body weight of mice across a 20-week study, with the control group (CTRL) on a standard diet and experimental groups on a HFD with varying interventions: low-dose SZA (HFD + SZA-L), high-dose SZA (HFD + SZA-H), and dapagliflozin (HFD + Dapa). Each data point is accompanied by an error bar indicating the standard error of the mean (SEM), showcasing the variability within each group. (**B**) illustrates the average daily food intake measured during the final week for each group, with error bars representing SEM. (**C**) displays the terminal intraperitoneal glucose tolerance test (IPGTT) results, measuring glucose levels at specific time intervals post-glucose administration, while (**D**) presents the intraperitoneal insulin tolerance test (IPITT) outcomes, tracking glucose levels at different time points after insulin injection. (**E**) depicts the terminal body weight of the mice with SEM error bars representing the variability in these measurements. Additionally, (**F**) shows the terminal fasting plasma glucose (FPG) levels, providing a snapshot of each group’s glucose metabolism under fasting conditions. Asterisks (*) denote significant differences compared to the control group (*p* < 0.05), and double asterisks (**) indicate highly significant differences compared to the control group (*p* < 0.01). Hashes (#) are used to show significant differences compared to the HFD group (*p* < 0.05).

To investigate the therapeutic effects of SZ-A on MAFLD, an experiment was completed with five groups of mice. The results showed that at week 20, mice receiving the HFD had a significantly higher body weight and more impaired glucose tolerance and insulin tolerance compared with the control group (CTRL). There was no significant difference in food consumption between all experimental groups. In HFD mice, after 8 weeks of drug intervention, mice in the SZ-A high-dose group had significantly inhibited HFD-induced weight gain and improved insulin tolerance (Figure 1A–D).

### 2.2. High-Dose SZ-A Significantly Reduced the Serum Liver Enzyme Concentrations and Improved Lipid Profiles in MAFLD Induced by HFD

**Figure 2:**
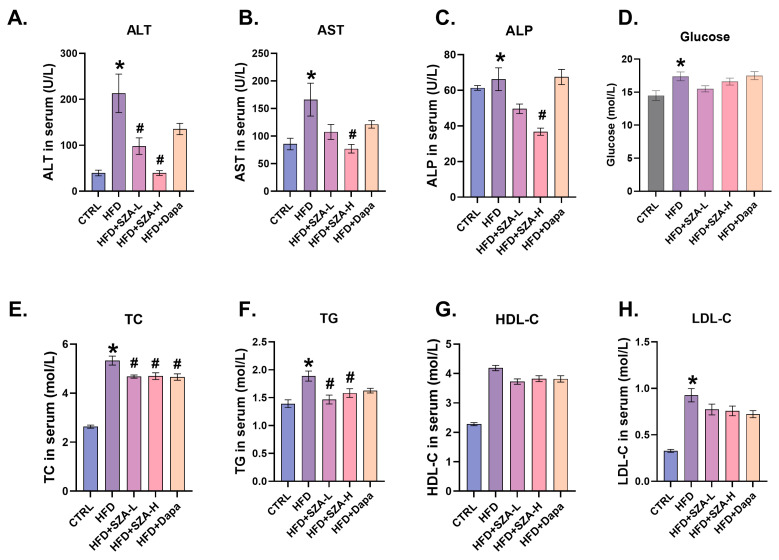
All comparisons of interventions were conducted at week 20. (**A**) The concentration of ALT in mouse serum (*n* = 10). (**B**) The concentration of AST in mouse serum (*n* = 10). (**C**) The concentration of ALP in mouse serum (*n* = 10). (**D**) The concentration of glucose in mouse serum (*n* = 10). (**E**) The concentration of TC in mouse serum (*n* = 10). (**F**) The concentration of TG in mouse serum (*n* = 10). (**G**) The concentration of HDL-C in mouse serum (*n* = 10). (**H**) The concentration of LDL-C in mouse serum (*n* = 10). The results of body weight change in mice (*n* = 10). Abbr: CTRL, control group; HFD, high-fat diet; SZ-A, Sangzhi-alkaloids; SZA-L, low-dose Sangzhi-alkaloids; SZA-H, high-dose Sangzhi-alkaloids; Dapa, dapagliflozin; ALT, alanine aminotransferase; AST, aspartate aminotransferase; ALP, alkaline phosphatase; TC, total cholesterol; TG, triglyceride; HDL-C, high-density lipoprotein cholesterol; LDL-C, low-density lipoprotein cholesterol. “*” means *p* < 0.05 compared to CTRL group, “#” means *p* < 0.05 compared to HFD group.

At week 20, compared with the CTRL group, the HFD group showed significantly increased levels of blood glucose, TG, TC, and LDL-C, as well as serum liver injury indexes including ALT, AST, and ALP. In HFD mice, after 8 weeks of high-dose SZ-A intervention, the serum ALT, AST, ALP, TC, and TG levels were significantly reduced, which were more prominent in the high-dose SZ-A group. In addition, there was a significant reduction in serum TC levels in the HFD + dapagliflozin group compared with the HFD group (Figure 2A–H).

### 2.3. SZ-A Could Improve Liver Hepatic Lipids in MAFLD Induced by HFD

**Figure 3:**
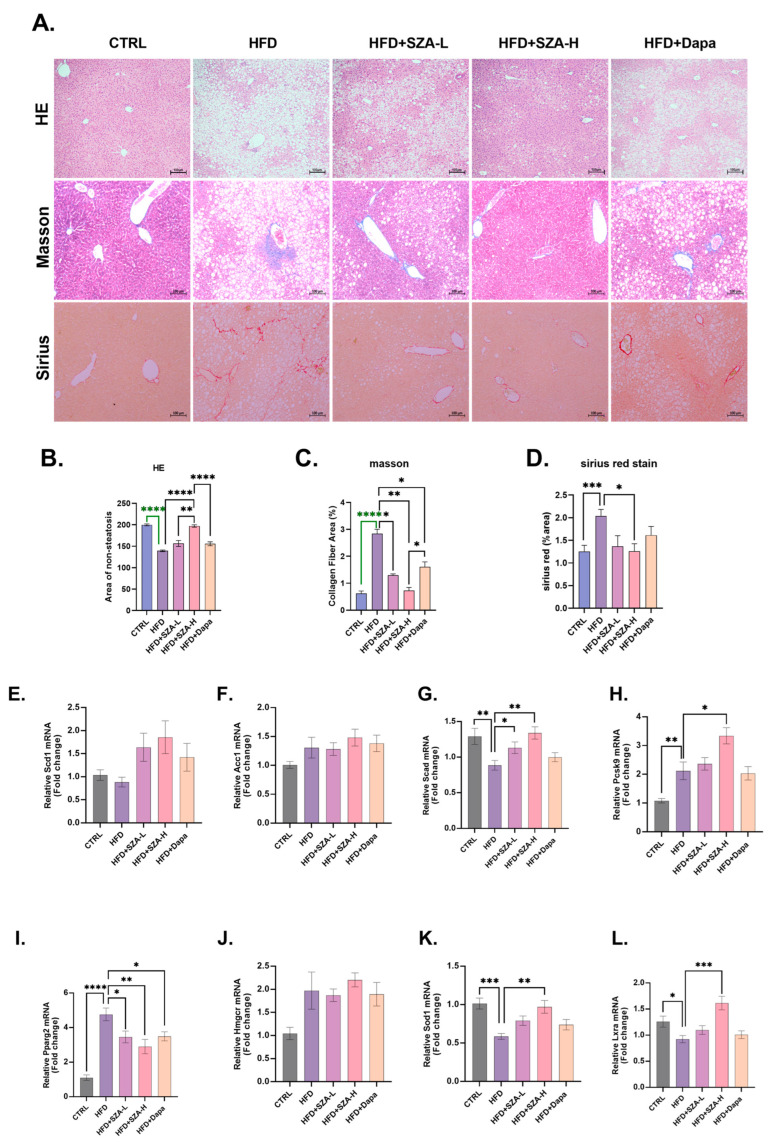

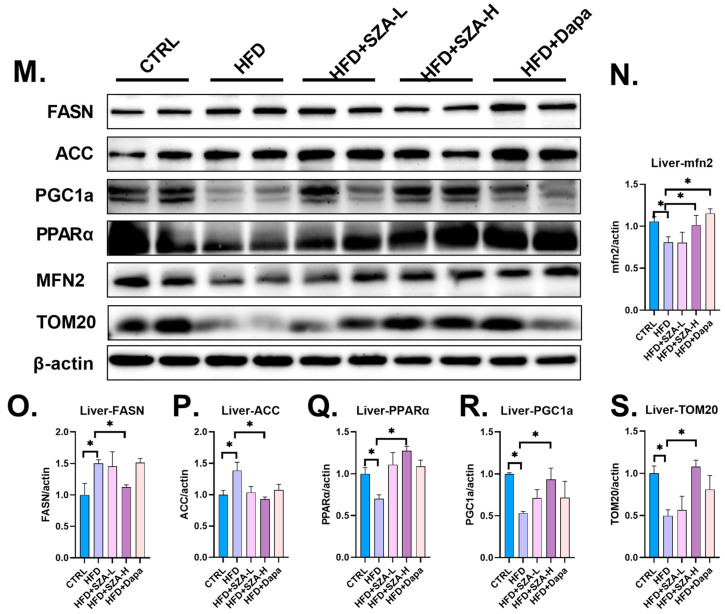
(**A**) Microscopic view of mouse liver after hematoxylin–eosin, Masson, and Sirius Red staining. (**B**) The results of hematoxylin–eosin (HE) staining of liver tissue of mice. (**C**) The results of Masson staining of liver tissue of mice. (**D**) The results of Sirius staining of liver tissue of mice. (**E**) The expression of SCD1 gene compared to GAPDH. (**F**) The expression of ACC1 gene compared to GAPDH. (**G**) The expression of SCAD gene compared to GAPDH. (**H**) The expression of PCSK9 gene compared to GAPDH. (**I**) The expression of PPARγ gene compared to GAPDH. (**J**) The expression of HMGCR gene compared to GAPDH. (**K**) The expression of SOD1 gene compared to GAPDH. (**L**) The expression of LXRα gene compared to GAPDH. (**M**) The result of immunoblotting. (**N**) The expression of MFN2 compared to β-actin. (**O**) The expression of FASN compared to β-actin. (**P**) The expression of ACC compared to β-actin. (**Q**) The expression of PPARα compared to β-actin. (**R**) The expression of PGC1α compared to β-actin. (**S**) The expression of TOM20 compared to β-actin. Abbr: CTRL, control diet; HFD, high-fat diet; SZA-L, low-dose Sangzhi-alkaloids; SZA-H, high-dose Sangzhi-alkaloids; Dapa, dapagliflozin; SCD1, stearoyl-CoA desaturase 1; ACC1, acetyl-CoA carboxylase 1; SCAD, short-chain acyl-coenzyme A dehydrogenase; PCSK9, proprotein convertase subtilisin/kexin type 9; PPARγ, peroxisome proliferator-activated receptor γ; HMGCR, 3-hydroxy-3-methylglutaryl-coenzyme A reductase; SOD1, superoxide dismutase-1; LXRα, liver X receptor α; MFN2, mitochondrial fusion protein; FASN, fatty acid synthase; ACC, acetyl-CoA carboxylase; PPARα, peroxisome proliferator-activated receptor α; PGC1α, peroxisome proliferator-activated receptor-γ coactivator 1α; TOM20, translocase of outer mitochondrial membrane 20. The symbols *, **, ***, and **** denote *p*-values of less than 0.05, 0.01, 0.001, and 0.0001, respectively. Note: The green color in (**B**,**C**) is used to distinguish annotations where lines overlap and does not indicate any specific meaning.

In the hematoxylin and eosin (HE) staining assessment of liver sections, we comparatively analyzed the non-steatotic regions among the different groups. The CTRL group exhibited a significantly higher area of non-steatosis compared to the HFD group (*p* < 0.0001). The HFD group demonstrated a significantly lower non-steatotic area compared to the high-dose SZ-A treatment group (HFD + SZA-H) (*p* < 0.0001), suggesting that high-dose SZ-A effectively ameliorates steatosis. Additionally, the low-dose SZ-A treatment group (HFD + SZA-L) had a significantly smaller non-steatotic area than the HFD + SZA-H group (*p* < 0.01), indicating a dose-dependent effect of SZ-A on reducing steatosis. Lastly, the HFD + SZA-H group exhibited a significantly higher area of non-steatosis compared to the dapagliflozin treatment group (HFD + Dapa) (*p* < 0.0001), suggesting that SZ-A has a greater advantage in reducing liver steatosis compared to dapagliflozin and indicating that the beneficial effects of SZ-A in reducing liver fat accumulation are independent of its glucose-lowering effects (Figure 3A,B).

We assessed the mRNA expression levels of genes implicated in hepatic lipid metabolism. In the HFD group, there was a significant upregulation of the mRNA levels for the genes PPGRγ and PCSK9, which are associated with lipid synthesis, compared to the CTRL group. Conversely, the mRNA levels of SCAD, SOD1, and LXRA, which are involved in lipid catabolism and antioxidant defense, were significantly downregulated in the HFD group. In comparison to the HFD group, the HFD + SZA-H group exhibited a significant decrease in PPGRγ mRNA levels. Additionally, the mRNA levels of SCAD, SOD1, and LXRA were significantly upregulated in the HFD + SZA-H group, suggesting an enhancement of lipid catabolism and antioxidant mechanisms. However, in contrast, the HFD + SZA-H group showed a significant increase in PCSK9 mRNA levels, which may suggest a complex regulation of lipid metabolism by SZA-H. These findings indicate that the SZA-H intervention can modulate the expression of key genes in lipid metabolism, promoting a shift towards more balanced lipid homeostasis in the liver (Figure 3E–L).

By Western blot analysis, the protein levels of key molecules involved in lipid synthesis, FASN and ACC, were significantly elevated in the HFD group relative to the CTRL group (*p* < 0.05), suggesting an upregulation of lipogenic pathways. In contrast, the protein levels of PGC1α, PPARα, MFN2, and TOM20, which are associated with lipid catabolism, were significantly reduced in the HFD group compared to the CTRL group (*p* < 0.05), indicating a downregulation of fatty acid oxidation mechanisms.

In the HFD + SZA-H group, treatment with high-dose SZ-A led to a significant decrease in the protein levels of FASN and ACC compared to the HFD group (*p* < 0.05), implying a reduction in lipid synthesis. Concurrently, there was a significant increase in the protein levels of PGC1α, PPARα, MFN2, and TOM20 in the HFD + SZA-H group compared to the HFD group (*p* < 0.05), which points towards an enhancement of lipid catabolism. These results (Figure 3M–S) demonstrate that high-dose SZ-A treatment can ameliorate the dysregulation of lipid metabolism in the liver by promoting fatty acid oxidation and inhibiting lipid synthesis.

### 2.4. SZ-A Improved Liver Fibrosis in MAFLD Induced by HFD and Affected the Expression of Oxidative Stress Kinase

In this study, we evaluated the effects of SZ-A on liver fibrosis and steatosis induced by HFD.

In the Masson staining analysis (Figure 3A,C), significant differences in collagen fiber areas were observed among the groups. The CTRL group demonstrated a significantly lower collagen fiber area compared to the HFD group (*p* < 0.0001), indicating a substantial reduction in fibrosis. The HFD group had a significantly higher collagen fiber area than both the HFD + SZA-L group (*p* < 0.05) and the HFD + SZA-H group (*p* < 0.01), suggesting that SZ-A treatment at both low and high doses effectively attenuates fibrosis. Furthermore, the HFD + SZA-H group showed a lower collagen fiber area than the HFD + Dapa group (*p* < 0.05), indicating that SZ-A may have a similar or greater effect in reducing fibrosis compared to dapagliflozin. The HFD + SZA-H group also had a significantly lower collagen fiber area than the HFD + Dapa group (*p* < 0.05), which further supports the potential therapeutic advantage of SZ-A in fibrosis reduction. These results are depicted in the figure.

In the Sirius Red staining evaluation, the CTRL group showed a significantly lower collagen fiber area compared to the HFD group (*p* < 0.001). The HFD + SZA-H group exhibited a significantly reduced collagen fiber area when compared to the HFD group (*p* < 0.05), indicating that the high-dose SZ-A treatment contributed to a decrease in collagen deposition. These results suggest that SZ-A may have a beneficial effect in reducing liver fibrosis as evidenced by the reduction in Sirius Red staining (Figure 3A,D).

In addition to the histological assessments, the protein levels of the liver fibrosis markers KEAP1 and α-SMA were significantly elevated in the HFD group compared to the CTRL group (*p* < 0.05), indicating increased fibrotic activity. Concurrently, the protein levels of NRF2 and its downstream antioxidant enzymes SCD1, GPX6, SOD1, and SOD2 were significantly decreased in the HFD group (*p* < 0.05), suggesting a reduction in the antioxidant defense mechanisms.

In contrast, the HFD + SZA-H group showed a significant reduction in the protein levels of KEAP1 and α-SMA compared to the HFD group (*p* < 0.05), indicating that high-dose SZ-A treatment effectively attenuated the fibrotic response. Furthermore, the protein expression levels of NRF2, SCD1, GPX6, SOD1, and SCD2 were significantly increased in the HFD + SZA-H group compared to the HFD group (*p* < 0.05), demonstrating an enhancement in the antioxidant capacity with high-dose SZ-A treatment.

**Figure 4:**
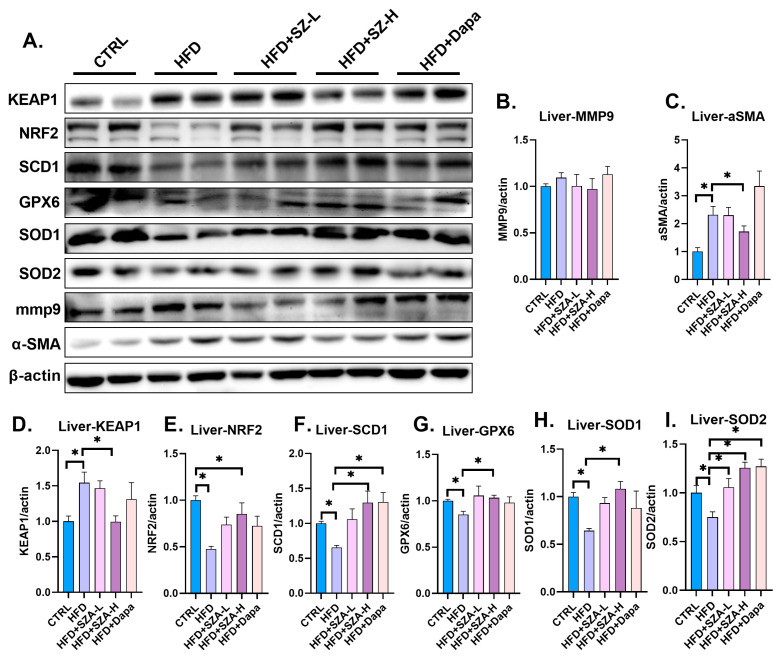
(**A**) The result of immunoblotting. (**B**) The expression of MMP9 compared to β-actin. (**C**) The expression of α-SMA compared to β-actin. (**D**) The expression of KEAP1 compared to β-actin. (**E**) The expression of NRF2 compared to β-actin. (**F**) The expression of SCD1 compared to β-actin. (**G**) The expression of GPX6 compared to β-actin. (**H**) The expression of SOD1 compared to β-actin. (**I**) The expression of SOD2 compared to β-actin. Abbr: CTRL, control diet; HFD, high-fat diet; SZA-L, low-dose Sangzhi-alkaloids; SZA-H, high-dose Sangzhi-alkaloids; Dapa, dapagliflozin; KEAP1, kelch-like ECH associated protein 1; MMP9, matrix metalloproteinase 9; α-SMA, alpha-smooth muscle actin; NRF2, nuclear factor erythroid-2-related factor 2; SCD1, stearyl coenzyme A dehydrogenase-1; GPX6, glutathione peroxidase 6; SOD1, superoxide dismutase-1; SOD2, superoxide dismutase-2. “*” means *p* < 0.05.

These findings (Figure 4A–I) indicate that high-dose SZ-A treatment effectively reverses the fibrotic and oxidative stress-induced changes in the liver.

### 2.5. SZ-A Improved Liver Lipid Metabolism by Upregulating the Synthesis of PGC1α

**Figure 5:**
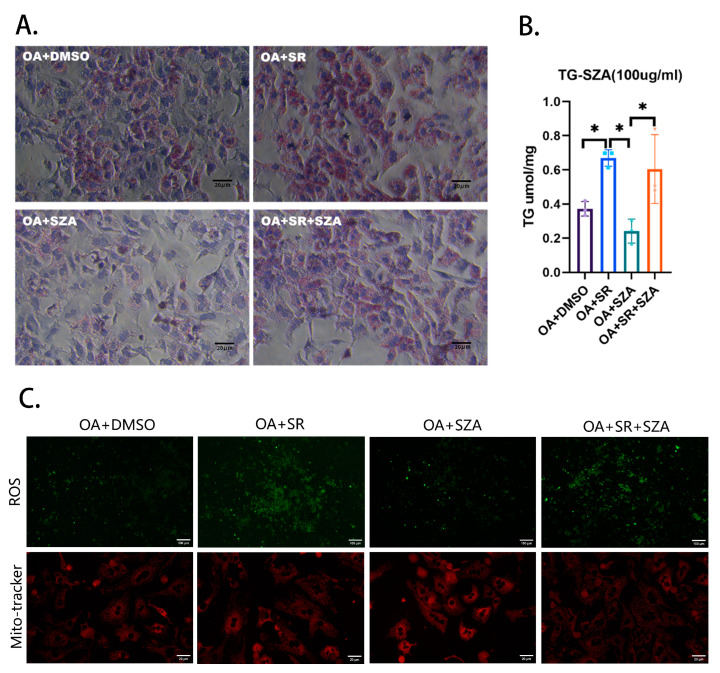
(**A**) The rescue experiment in hepatocytes. (**B**) The content of TG in hepatocytes (*n* = 3). (**C**) The content of ROS and mitochondria in hepatocytes (*n* = 3). Abbr: SZA, Sangzhi-alkaloids; Dapa, dapagliflozin; OA, oleic acid; DMSO, dimethyl sulfoxide; SR, SR18292. TG, triglyceride; ROS, reactive oxygen species. “*” means *p* < 0.05.

To investigate the role of SZ-A in lipid metabolism, potentially through the regulation of the PGC1α molecule, we performed a rescue experiment using primary liver cells from wild-type mice. The groups are as follows: OA + DMSO serving as the high-lipid control group, OA + SR representing the group treated with oleic acid and the PGC1α inhibitor SR18292, OA + SZA indicating the group treated with oleic acid and SZ-A, and OA + SR + SZA for the group treated with all three agents. The specific values for the TG content are illustrated in the figure, with Figure 5A showing the Oil Red O staining results and Figure 5B displaying the TG content measurements.

We observed that compared to the high-lipid control group (OA + DMSO), the TG content in the primary liver cells of the OA + SR group was significantly increased. The addition of SZ-A to the OA + SZA group led to a significant decrease in the TG content, suggesting that SZ-A can effectively reduce lipid accumulation. However, the TG-lowering effect of SZ-A was less pronounced when the PGC1α inhibitor SR was also present, as indicated by the TG content in the OA + SR + SZA group.

Additionally, in the rescue experiment, compared to the control group, treatment of hepatocytes with the PGC1α inhibitor resulted in an increase in the intracellular ROS content and a decrease in the number of intracellular mitochondria, while SZ-A treatment of hepatocytes resulted in an increase in the intracellular mitochondria number (Figure 5C).

### 2.6. Adiponectin and Leptin Levels Were Not Significantly Affected by SZ-A

**Figure 6:**
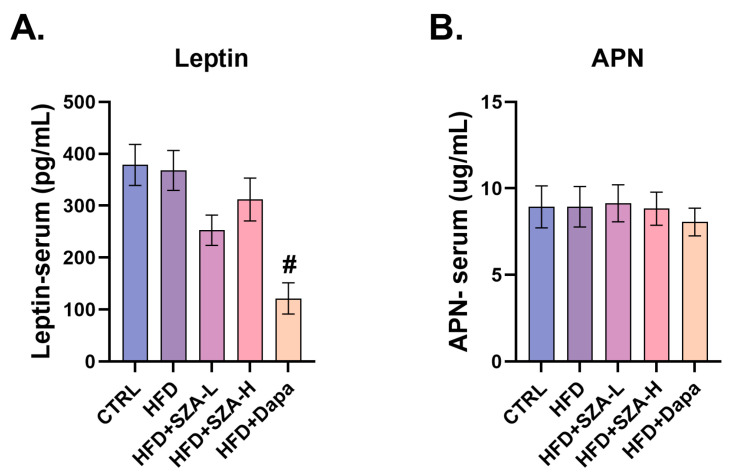
(**A**) The concentration of leptin in mouse serum (*n* = 10). (**B**) The concentration of APN in mouse serum (*n* = 10). Abbr: CTRL, control group; HFD, high-fat diet; SZA-L, low-dose Sangzhi-alkaloids; SZA-H, high-dose Sangzhi-alkaloids; Dapa, dapagliflozin; APN, adiponectin. “#” means *p* < 0.05 compared to HFD group.

It was deemed that HFD might also cause disorders in adipocytokine secretion by adipose tissue. To further address this issue, we measured the serum levels of adiponectin (APN) and leptin. However, we did not find a significant effect of SZ-A on serum APN and leptin levels (Figure 6A,B).

### 2.7. PGC1α Might Interact with NRF2 at the Molecular Level

**Figure 7:**
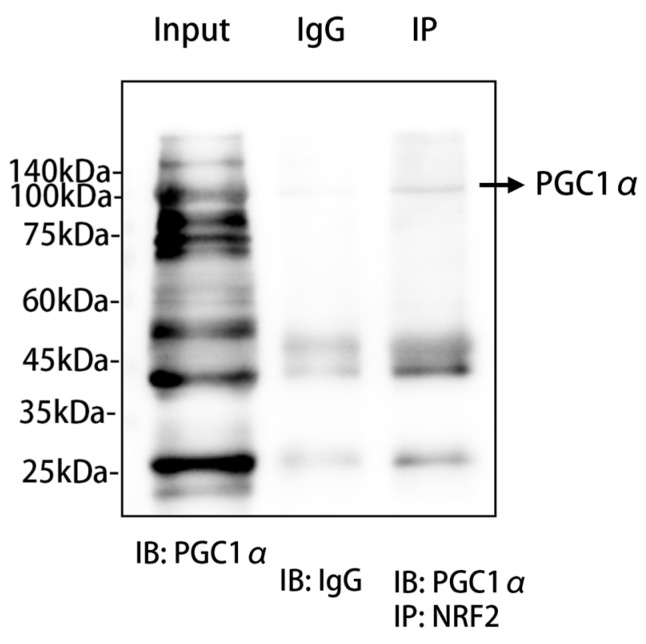
PGC1α and NRF2 co-immunoprecipitation experiments. We conducted co-immunoprecipitation (Co-IP) experiments using primary hepatocytes from mice to explore the potential interaction between NRF2 and PGC1α proteins. The experimental results are depicted in the figure, which includes the input group showing the baseline expression of PGC1α protein in whole-cell lysates from primary mouse hepatocytes without immunoprecipitation treatment; the IP group, where protein complexes interacting with NRF2 were precipitated using a specific antibody against NRF2; and the negative control group, which used non-specific IgG antibody precipitation to confirm specific binding during the precipitation process. The figure also includes molecular weight markers ranging from 140 kDa to 25 kDa to estimate the molecular weight of the protein bands. Western blot technology was employed, utilizing a specific antibody against PGC1α (IB: PGC1α) and a non-specific IgG antibody (IB: IgG) to detect and verify the specificity of the precipitation. All experimental procedures were carried out under strict conditions to ensure the accuracy and reproducibility of the results. Abbr: PGC1α, peroxisome proliferator-activated receptor-γ coactivator 1α; IgG, immunoglobulin G; NRF2, nuclear factor erythroid-2-related factor 2; IP, immunoprecipitation; IB, immunoblotting.

In order to assess the association between PGC1α and NRF2, we used co-immunoprecipitation as a method to investigate the potential interaction between them. PGC1α was present in the immunoprecipitated samples, while the control experiments using non-specific IgG antibodies did not show significant co-immunoprecipitation, which provided reciprocal evidence for the interaction between PGC1α and NRF2 (Figure 7).

## 3. Discussion

In this study, we established an MAFLD mouse model by feeding male C57BL/6J mice with 60% HFD for 12 weeks and observed the progression and prognosis of MAFLD after 8 weeks of drug intervention (including SZ-A at low or high doses, dapagliflozin). The results indicated that in HFD mice, all low-dose SZ-A, high-dose SZ-A, and dapagliflozin interventions significantly improved insulin tolerance, lowered blood lipids and ameliorated liver steatosis and liver fibrosis in HFD mice compared with the intervention of normal saline, where the effect of high-dose SZ-A was better than low-dose SZ-A and dapagliflozin. The mechanism by which SZ-A improves the lipid metabolism in MAFLD model mice might be related to the regulation of PGC1α-PPARα/PPARγ. In addition, the mechanism of SZ-A ameliorating oxidative stress and hepatic fibrosis in MAFLD model mice might be associated with PGC1α-induced KEAP1/NRF2 axis activation.

### 3.1. SZ-A Improved Liver Lipid Metabolism in MAFLD Mouse Models

Obesity, which leads to the development of metabolic syndrome and multiple comorbidities, has become a global health problem [14]. With the rising trend in obesity, the prevalence and severity of MAFLD are also increasing worldwide [4]. At present, the targeted management of obesity through lifestyle interventions remains the cornerstone of the treatment of MAFLD [15]. In addition to that, medications to ameliorate MAFLD were also considered to be an important method of therapy. An increasing number of studies were conducted to find effective agents to alleviate MAFLD through multiple mechanisms. Our results showed that high-dose SZ-A treatment could significantly reduce the body weight, decrease the serum lipids, improve insulin tolerance, and ameliorate liver steatosis and fibrosis in MAFLD model mice. The body-weight-lowering effects of high-dose SZ-A were independent of food intake, blood glucose, and glucose tolerance, suggesting that SZ-A might reduce body weight through the regulation of molecules involved in the lipid metabolism pathway.

In previous studies, PGC1α was also considered an important therapeutic target for type 2 diabetes and obesity through regulating mitochondrial biogenesis and interacting with transcription factors such as estrogen-related receptors, liver X receptors, and hepatic nuclear factor 4α to coordinate the expression of mitochondrial genes and indirectly promote the transport and utilization of fatty acids (FAs), thereby regulating glucolipid metabolism [16,17,18]. Meanwhile, PGC1α could also activate the function of PPARα and PPARγ [19,20]. PPARα is mainly expressed in the liver and controls many intracellular processes involved in lipid metabolism, including peroxisome reactions and mitochondrial fatty acid oxidation (FAO) [19]. In contrast, PPARγ is widely expressed in liver cells and adipose tissues, which mainly function in regulating adipocyte differentiation. The incitation of PPARγ could also increase the proportion of brown adipose tissues and facilitate energy consumption [20]. In previous studies, it was also observed that the overexpression of PGC1α in the liver significantly increased hepatic FA oxidation and decreased TG storage and secretion both in vivo and in vitro [17], indicating the therapeutic potential of PGC1α in improving MAFLD conditions through the abovementioned mechanisms.

These are consistent with our findings. We found that the HFD decreased the expression of PGC1α and its downstream molecule PPARα in the liver, whereas high-dose SZ-A upregulated the expression of PGC1α and PPARα in the liver. At the same time, our rescue experiment in primary mouse liver cells further demonstrated that PGC1α may serve as a key element for the lipid metabolism regulation effects of SZ-A. In other words, the improvement in MAFLD by SZ-A treatment was potentially achieved by upregulating PGC1α synthesis in the liver.

### 3.2. SZ-A Improved Liver Fibrosis and Oxidative Stress in MAFLD Model Mice

MAFLD caused by HFD could not only induce simple hepatic steatosis but also cause oxidative stress in hepatocytes and liver fibrosis, which mutually promote the progression of MAFLD itself, eventually leading to liver cirrhosis and hepatocellular carcinoma. According to our study, it was suggested that SZ-A could reduce body weight, alleviate lipid metabolism profiles, decrease indices reflecting liver injury, and retard liver pathological progression in mice with MAFLD. Furthermore, the MAFLD therapeutic effects of SZ-A were believed to be associated with the activation of several substances as mentioned above. Moreover, relevant studies also suggested that SZ-A could activate the NRF2 axis to inhibit the excitation of hepatic stellate cells, thereby alleviating liver fibrosis [21,22]. It was also observed that PGC1α could regulate the expression of nuclear/mitochondrial genes associated with oxidative phosphorylation by coactivating NRF2, indicating that NRF2 may also act as an important pathway through which SZ-A exerts MAFLD-remedying effects [23].

The KEAP1/NRF2/ARE axis plays a major role in the regulation of cellular redox balance. NRF2 and its endogenous inhibitor KEAP1, as a universal regulator of intracellular defense mechanisms, often fight against oxidative stress in the body [24]. Under normal conditions, KEAP1 binds to NRF2 and targets it to accelerate proteasome degradation and the regeneration of KEAP1 [25]. However, in the case of oxidative stress, the interaction between NRF2 and KEAP1 is interrupted, the contents of NRF2 are increased, and the synthesis of downstream antioxidant molecules, such as heme oxygenase-1 (HO-1), glutathione-S-transferase (GST), glutathione peroxidase (GPX), NAD(P)H quinone oxidoreductase 1 (NQO1), superoxide dismutase (SOD), catalase (CAT), and glutathione reductase (GR), is reduced [26]. Therefore, NRF2-related pathways have the potential to improve MAFLD through mechanisms including liver fibrosis suppression and oxidative stress inhibition.

Our results indicated that high-dose SZ-A could activate the inhibited NRF2 (by HFD), thereby upregulating the expression of the downstream antioxidant molecules SOD1 and GPX6 and repressing the expression of the liver fibrosis molecule α-SMA. We also found that PGC1α might be coactivated with NRF2 to play a downstream activation role. It was suggested that SZ-A might counteract liver oxidative stress and liver fibrosis induced by HFD through the KEAP1/NRF2/ARE axis.

In addition, SCD1 is a key enzyme in the production of fat in the liver, which converts saturated fatty acids into monounsaturated fatty acids. When SCD1 is inhibited, fatty acid synthesis is restrained and beta-oxidation is increased, resulting in decreased TG storage in the liver [27]. However, previously, no systemic manifestations of lipid reduction were observed in liver-specific SCD1 gene knockout animal models [28]. SCD1 inhibition was shown to directly reduce the levels of the antioxidant enzyme GPX4 and the ratio of reduced glutathione/oxidized glutathione (GSH/GSSG), which promoted the increase in intracellular reactive oxygen species (ROS) and led to mitochondrial redox imbalance, intracellular lipid peroxidation, and mitochondrial dysfunction [29].

In our study, we found that HFD significantly reduced the contents of SCD1, while high-dose SZ-A treatment significantly upregulated SCD1 synthesis, suggesting that SCD1 may have a role mainly in antioxidant stress rather than in the promotion of fatty acid synthesis in the liver.

### 3.3. Summary

To summarize, based on the study by Morieri, M [30], it can be inferred that dapagliflozin may indirectly regulate hepatic steatosis by affecting blood glucose levels. To exclude the possibility that SZ-A also indirectly affects hepatic steatosis through blood glucose-lowering effects, we conducted a drug control experiment using dapagliflozin. Our results showed that, compared to dapagliflozin, SZ-A more significantly reduced hepatic steatosis in mouse liver tissue. Furthermore, when we treated primary hepatocytes from mice with SZ-A, we observed a notable decrease in the triglyceride content in the liver cells treated with higher doses of the alkaloids. Thus, we can reasonably infer that SZ-A can reduce the lipid content in hepatocytes independent of its blood glucose-lowering effects.

In our study, we investigated whether the protective effect of SZ-A on MAFLD mice is independent of its effect on body weight. We also observed the phenotype that occurs when SZ-A targets hepatocytes and attempted to explore the mechanism by which SZ-A acts directly on hepatocytes. Our experimental results suggested that by regulating the expression of PGC1α and its interacting KEAP1/NRF2 pathway in mouse liver cells, SZ-A played important roles in regulating lipid metabolism, inhibiting oxidative stress, and postponing liver fibrosis in mice with MAFLD as aforementioned.

## 4. Materials and Methods

### 4.1. Establishment of Animal Models

This study was ethically approved by the Institutional Animal Care and Use Committee (IACUC) of Peking University People’s Hospital (No. 2021PHE022). All protocols were executed in compliance with the directives outlined in the National Institutes of Health (NIH) Guide for the Care and Use of Laboratory Animals [31].

Beijing Wehand-Bio Pharmaceutical Co., Ltd. (Beijing, China) generously supplied Ramulus Mori (Sangzhi) alkaloids (SZ-A) powder (lot number: J202107010). The SZ-A powder contains 60.62% total polyhydroxy alkaloids, with specific contents of 40.75% DNJ, 8.99% FA, and 8.59% DAB [10].

The mice were kept in an environment maintained at a steady temperature of 26 °C, following a 12 h light and 12 h dark cycle. Each cage housed 3–5 mice and provided unrestricted access to food and water. Eight-week-old male C57BL/6J mice were fed with CTRL or 60% HFD for 12 weeks to establish normal mouse models and diet-induced obesity mouse models. From the 13th week, the HFD mice were administered SZ-A (divided into two dose groups: 400 mg/kg/d as HFD plus low-dose SZ-A, and 800 mg/kg/d as HFD plus high-dose SZ-A; SZ-A was dissolved in normal saline; *n* = 10 for each group) or the sodium glucose cotransporter-2 (SGLT-2) inhibitor dapagliflozin (1 mg/kg/d, namely HFD plus dapagliflozin; dapagliflozin was dissolved in normal saline; *n* = 10), and an equivalent normal saline (HFD group, *n* = 10) was included. In addition to this, there was a control diet group (abbreviated as CTRL, *n* = 10) that was observed until the 20th week. Fasting blood glucose and glucose and insulin tolerance were measured at baseline and before the end of the experiment. The weight and food intake of the mice were measured every week during the treatment process. At week 20, the mice were sacrificed, and all necessary samples were acquired. After fasting for 12 h, blood was taken from the heart of the mice. The liver and adipose tissue (including the visceral fat mass, perirenal adipose tissue, and epididymal adipose tissue) were cut, rinsed, weighed, and placed in the refrigerator at −80 °C for subsequent determination of relevant measurements.

### 4.2. Serological Tests

Commercial kits were used to determine the contents of serum glucose, total cholesterol (TC) (BioVision Research Products, Inc., Milpitas, CA, USA), triglyceride (TG), free fatty acids (FFAs) (Wako Chemicals, Neuss, Germany), aspartate aminotransferase (AST), alanine aminotransferase (ALT), alkaline phosphatase (ALP), high-density lipoprotein cholesterol (HDL-C), low-density lipoprotein cholesterol (LDL-C) (Cayman Chemical Company, Ann Arbor, MI, USA), adiponectin, and leptin (Solarbio, Beijing, China).

### 4.3. Histological Examination

The liver tissues were stained with hematoxylin-eosin (HE), Masson trichrome, and Sirius Red to observe the degrees of liver morphological changes, hepatic steatosis, and hepatic fibrosis.

### 4.4. Gene and Protein Level Detection

We examined the expression levels of genes and proteins related to lipid metabolism in mouse liver tissues.

The mRNA and protein levels of adipogenesis-related factors were detected, including fatty acid synthase (FASN), acetyl-CoA carboxylase (ACC) peroxisome proliferator-activated receptor γ (PPARγ), peroxisome proliferator-activated receptor α (PPARα), peroxisome proliferator-activated receptor-γ coactivator 1α (PGC1α), liver X receptor α (LXRα), nuclear factor erythroid-2-related factor 2 (NRF2), short-chain acyl-coenzyme A dehydrogenase (SCAD), stearyl coenzyme A dehydrogenase-1 (SCD1), superoxide dismutase-1 (SOD1), superoxide dismutase-2 (SOD2), glutathione peroxidase 6 (GPX6), matrix metalloproteinase 9 (MMP9), proprotein convertase subtilisin/kexin type 9 (PCSK9), 3-hydroxy-3-methylglutaryl-coenzyme A reductase (HMGCR), mitochondrial fusion protein (MFN2), translocase of outer mitochondrial membrane 20 (TOM20), and alpha-smooth muscle actin (α-SMA). Meanwhile, we used the co-Immunoprecipitation kit (Absin, Shanghai, China) to detect whether PGC1α interacts with NRF2. All antibodies were sourced from Abmart Shanghai Co., Ltd. (Shanghai, China)

### 4.5. Isolation of Murine Hepatocytes

We used the protocols developed by Malar et al., 2007 [32] for the isolation of hepatocytes. We took the liver from the animal and placed it in a sterile petri dish containing cold phosphate buffered saline (PBS). The liver was then dissected into small pieces using sterile instruments and transferred to a suitable digestive solution in a petri dish. After the liver fragments were incubated at the appropriate temperature for a period, they were digested using pancreatic enzymes. The cell suspension was then filtered with a sterile strainer or filter to remove undigested tissue and obtain a single-cell suspension. Cell suspensions were washed with a culture medium to neutralize enzymes and remove debris. The cell suspension was centrifuged, and the liver cells were collected at the bottom of the tube. The liver cells were suspended in a suitable medium. Finally, the cells were placed in a petri dish to promote cell attachment and growth. All consumables were purchased from Solarbio Science & Technology Co., Ltd. (Beijing, China)

### 4.6. Rescue Experiment of Liver Primary Cells

The liver cells of wild-type C57BL/6J mice were collected for the rescue experiment. Oleic acid (OA) and palmitic acid (PA) were added to 1640 complete medium at a ratio of 200:100 to simulate a high-lipid environment, where the liver primary cells in 12-well plates were divided into 4 treatment groups: high-fat group (oleic acid [OA] + dimethyl sulfoxide [DMSO]); high-fat + PGC1α inhibitor group (SR18292, dissolved with DMSO, concentration 25 μM) (OA + SR); high-fat + SZA group (OA + SZA, concentration 100 μM); high-fat + SR + SZA group (OA + SR + SZA). After being processed with the above treatments for 48 h, the cells were collected for Oil Red O staining and cell TG content detection. Kits measuring TG, reactive oxygen species (ROS), and mito-tracker were purchased from Solaibao Technology company (Beijing, China). Other reagents and consumables were purchased from Beyotime Biotechnology (Shanghai, China).

### 4.7. Statistical Analysis

The analysis of protein expression and adipocyte area was conducted using Image J software, version 1.53t (NIH, Bethesda, MD, USA). Graphs illustrating the results were generated with Prism 8 software (GraphPad Software Inc., San Diego, CA, USA). Statistical analyses were implemented with IBM SPSS Version 26.0. Statistical differences between two groups were examined by Student’s *t*-test. Analysis of variance (ANOVA) and multiple comparisons were applied for the comparisons among more than two groups. The results are presented in the format of mean ± standard error of the mean (SEM). Statistical significance was considered at *p* < 0.05.

## 5. Conclusions

In this study, we found that treatment with SZ-A could promote hepatic cellular lipid metabolism, reduce hepatic lipid deposition, alleviate hepatic oxidative stress, and attenuate hepatic fibrosis. The key lipid metabolism (PGC1a/PPARγ) and antioxidant stress (KEA2/NRF2) pathways played essential roles in the function of SZ-A in alleviating MAFLD progression. We also preliminarily observed a potential interaction between PGC1a and NRF2, which also suggested that lipid metabolism and oxidative stress were mutually complementary in MAFLD and that SZ-A was likely to be an effective drug to simultaneously improve lipid metabolism and oxidative stress.

## Data Availability

All data are provided in the original text.
